# Ferroptosis of smooth muscle cells in vascular diseases: from basic principles to clinical translation

**DOI:** 10.1038/s41420-026-02950-1

**Published:** 2026-03-09

**Authors:** Yiqing Yang, Abdul Qadir Nawabi, Yuyu Yao, Naifeng Liu

**Affiliations:** Department of Cardiology, Zhongda Hospital, Southeast University, Nanjing, China

**Keywords:** Vascular diseases, Cell death

## Abstract

Vascular smooth muscle cells (VSMCs), which form the media layer of blood vessels, play a vital role in vascular homeostasis and remodeling. Dysfunction of VSMCs represents a key pathological basis and an important contributor to vascular diseases. Ferroptosis, an iron-dependent accumulation of lipid hydroperoxides, is a novel form of regulated cell death. VSMC ferroptosis is involved in a range of vascular diseases, such as atherosclerosis, vascular calcification, hypertension, aortic aneurysm, aortic dissection, neointimal hyperplasia, intracranial aneurysm, and pulmonary arterial hypertension. This review summarizes the current evidence, underlying potential mechanisms, and therapeutic targets of VSMC ferroptosis in vascular diseases. A deeper understanding of this process may provide therapeutic insights and help in mitigating cardiovascular risk in affected patients.

## Facts

- Vascular diseases, significantly impair patients’ quality of life and pose a serious threat to their health and survival.
- Vascular smooth muscle cells (VSMCs) play essential roles in the maintenance of vascular homeostasis.
- Ferroptosis, an iron-dependent accumulation of lipid hydroperoxides, is a novel form of regulated cell death.
- VSMCs ferroptosis is one of the multiple mechanisms which contribute to vascular diseases.

## Open questions

- What are the precise molecular mechanisms by which VSMC ferroptosis contributes to the progression of vascular diseases?
- What are the established and emerging mechanisms of ferroptosis, and how do they contribute to understanding and addressing various diseases?
- Could targeting VSMCs offer therapeutic strategies to mitigate ferroptosis-driven vascular diseases such as atherosclerosis, aortic aneurysm, and pulmonary hypertension?

## Introduction

Vascular diseases, primarily resulting from vascular lesions within the circulatory system, significantly impair patients’ quality of life and pose a serious threat to their health and survival. In recent years, vascular diseases have become the leading cause of mortality worldwide [1]. Vascular diseases can be specifically divided into three main categories: cardiovascular diseases (atherosclerosis, vascular calcification, hypertension, ischemia reperfusion injury, aortic aneurysm (AA), aortic dissection and neointimal hyperplasia), cerebrovascular diseases (intracerebral hemorrhage, subarachnoid hemorrhage, stroke and intracranial aneurysm; IA), pulmonary vascular diseases (acute lung injury, pulmonary hypertension). These vascular diseases exhibit high global morbidity and mortality, and are widely recognized as a major public health concern [2]. The development of novel therapeutic strategies remains both a research priority and a persistent challenge in the field.

Vascular smooth muscle cells (VSMCs) constitute a predominant cell type within the medial layer of the vascular wall. They play an essential role in regulating vascular remodeling and maintaining vascular homeostasis. Recent studies have demonstrated remarkable phenotypic plasticity of VSMCs. In response to vascular injury or stimulation by cytokines and growth factors, VSMCs can transition from a contractile to a synthetic phenotype. This phenotypic switch is characterized by enhanced proliferation, reduced contractile function, and secretion of matrix-degrading enzymes, ultimately contributing to vascular remodeling [3, 4].

Ferroptosis, an iron-dependent accumulation of lipid hydroperoxides, is a novel form of regulated cell death which was first described in 2012 [5]. During this process, ferroptotic cells display shrunken mitochondria, exhibiting reduced or absent cristae, increased membrane density, and rupture of the outer mitochondrial membrane. Numerous intracellular molecules regulate ferroptosis by modulating iron homeostasis and the extent of lipid peroxidation. Furthermore, ferroptosis has been implicated in a wide range of pathological processes, including degenerative diseases, cancer, and organ injury, warranting further in-depth investigation.

The vascular wall is primarily composed of endothelial cells [6], smooth muscle cells, and fibroblasts [7], all of which may contribute to the initiation and progression of ferroptosis. However, this review focuses specifically on ferroptosis in VSMCs, given their central role in vascular remodeling and disease pathogenesis. VSMCs ferroptosis, is one of the multiple mechanisms which lead to vascular diseases. Recent studies suggest that inhibition of ferroptosis can alleviate vascular diseases [8, 9]. Therefore, targeting ferroptosis may offer a promising therapeutic approach for protecting VSMCs and preventing vascular injury. This review summarises the fundamental features of ferroptosis, discusses the role of VSMC ferroptosis in various vascular diseases, and highlights the emerging anti-ferroptosis therapeutic strategies, which may represent future research trends and focal points in this field.

## Ferroptosis: mechanism and regulatory pathways

Ferroptosis is primarily driven by intracellular iron accumulation and lipid peroxidation. Even before the concept of ferroptosis was formally proposed, studies had demonstrated that treatment with iron chelators or reduction of intracellular iron levels could significantly suppress this form of cell death [10]. Owing to its distinctive dependence on iron, this form of cell death was termed “ferroptosis” in 2012 [5]. Numerous factors influence intracellular ferroptosis by regulating iron transport and metabolism [11]. Divalent metal transporter 1 (DMT1) can transport divalent metal ions, including iron ions. Song et al. reported that DMT1 expression is significantly upregulated in an acute myocardial infarction model. Notably, overexpression of DMT1 enhances hypoxia/reoxygenation-induced ferroptosis in cardiomyocytes, whereas its downregulation markedly suppresses this process [12].

**Fig. 1:**
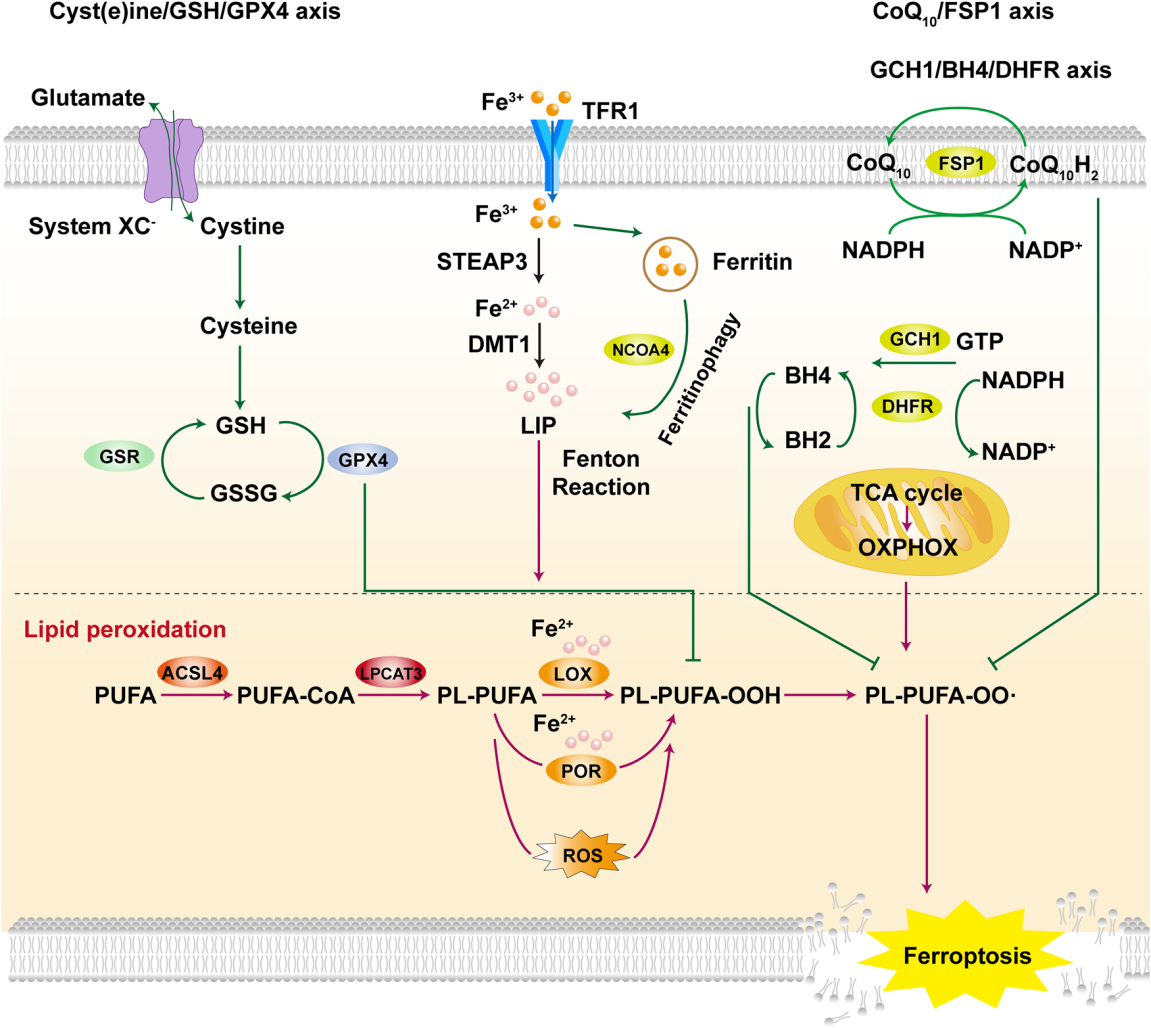
Schematic description of the signaling pathway in ferroptosis. Iron is transported by transferrin receptor (TFR1) as Fe^3+^. Fe^3+^ is reduced to Fe^2+^ by STEAP3 metalloreductase (STEAP3) and transferred to the cytoplasm through divalent metal transporter 1 (DMT1). Triggered by low intracellular iron levels, ferritinophagy is induced by nuclear receptor coactivator 4 (NCOA4). This leads to Fe^2+^ overload and activates the Fenton reaction, which produces large reactive oxygen species (ROS) and mediates lipid peroxidation. Besides, another important factor contributing to ferroptosis is impaired antioxidant capacity. For example, cyst(e)ine/glutathione (GSH)/GPX4 axis is a classic ferroptosis pathway. GPX4 can inhibit ferroptosis by reducing ROS production and lipid peroxidation. Moreover, CoQ_10_/FSP1 axis, GCH1/BH4/DHFR axis and mitochondrial energy metabolism are involved in the signaling pathway in ferroptosis.

Another key factor that contributed to ferroptosis is membrane lipid peroxidation. Acyl-CoA synthetase long-chain family member 4 (ACSL4) is a lipid metabolism-related gene that encodes an enzyme critical for the esterification of polyunsaturated fatty acids (PUFAs) and their incorporation into membrane phospholipids [13, 14]. Phospholipids incorporating PUFAs contain unstable bis-allylic hydrogen atoms, making them highly susceptible to lipid peroxidation, which in turn drives the execution of ferroptosis [15]. Glutathione peroxidase 4 (GPX4) is a key regulator of ferroptosis. It reduces lipid hydroperoxides to non-toxic lipid alcohols, thereby suppressing lipid peroxidation and preventing the onset of ferroptosis [16]. Park et al. reported that inhibition of GPX4 leads to lipid peroxide accumulation in H9c2 cells and triggers ferroptotic cell death [17]. Ferroptosis suppressor protein 1 (FSP1), also known as apoptosis-inducing factor mitochondria-associated 2 (AIFM2), functions independently of GPX4 to inhibit lipid peroxidation and thereby attenuate ferroptosis [18, 19]. FSP1 reduces coenzyme Q10 (CoQ10) to its active form, ubiquinol (CoQ_10_H₂), a lipophilic antioxidant that scavenges free radicals and suppresses lipid peroxidation, thereby inhibiting ferroptosis [20]. A schematic description of the signaling pathway in ferroptosis is depicted in Fig. 1.

## VSMCs dysfunction

VSMCs are the predominant component of the arterial medial layer and play a pivotal role in maintaining vascular integrity and function. Their primary physiological functions include contraction, regulation of vascular tone, control of blood flow, and maintenance of blood pressure. In addition to their contractile function, VSMCs possess biosynthetic and proliferative capabilities that contribute to vessel wall homeostasis [21]. Fully differentiated or mature VSMCs exhibit a contractile phenotype characterized by a relatively low proliferative capacity and the expression of specific contractile proteins, such as smooth muscle myosin heavy chain, smooth muscle 22α (SM22α), and calmodulin [22], which are essential for sustaining contractile activity.

Under various pathological conditions, dysfunction of VSMCs, often resulting from VSMC injury or death, is closely associated with adverse clinical outcomes. For example, in response to vascular injury induced by procedures such as angioplasty or bypass surgery, VSMCs undergo a phenotypic switch from a contractile to a synthetic state. This synthetic phenotype is characterized by enhanced proliferation, migration, and extracellular matrix (ECM) production, along with the downregulation of VSMC-specific markers [23]. The transition to the synthetic phenotype contributes to the progression of atherosclerosis, hypertension, and neointima formation [21, 24].

Increasing evidence indicates that VSMC phenotypic modulation is also implicated in other vascular pathologies, including vascular calcification [25, 26], AA [27], and pulmonary hypertension [28]. Moreover, various forms of VSMC death such as apoptosis [29, 30], necroptosis [31, 32], pyroptosis [33, 34] and ferroptosis [35, 36] have been implicated in the development of VSMC dysfunction. However, the precise underlying mechanisms of these processes remain to be fully elucidated. Further investigation is required to clarify the distinct roles and regulatory pathways of each cell death modality, which may provide novel therapeutic targets to promote VSMC survival under pathological conditions and facilitate the development of effective treatments for VSMC-related vascular disorders.

## Ferroptosis and specific vascular diseases

**Fig. 2:**
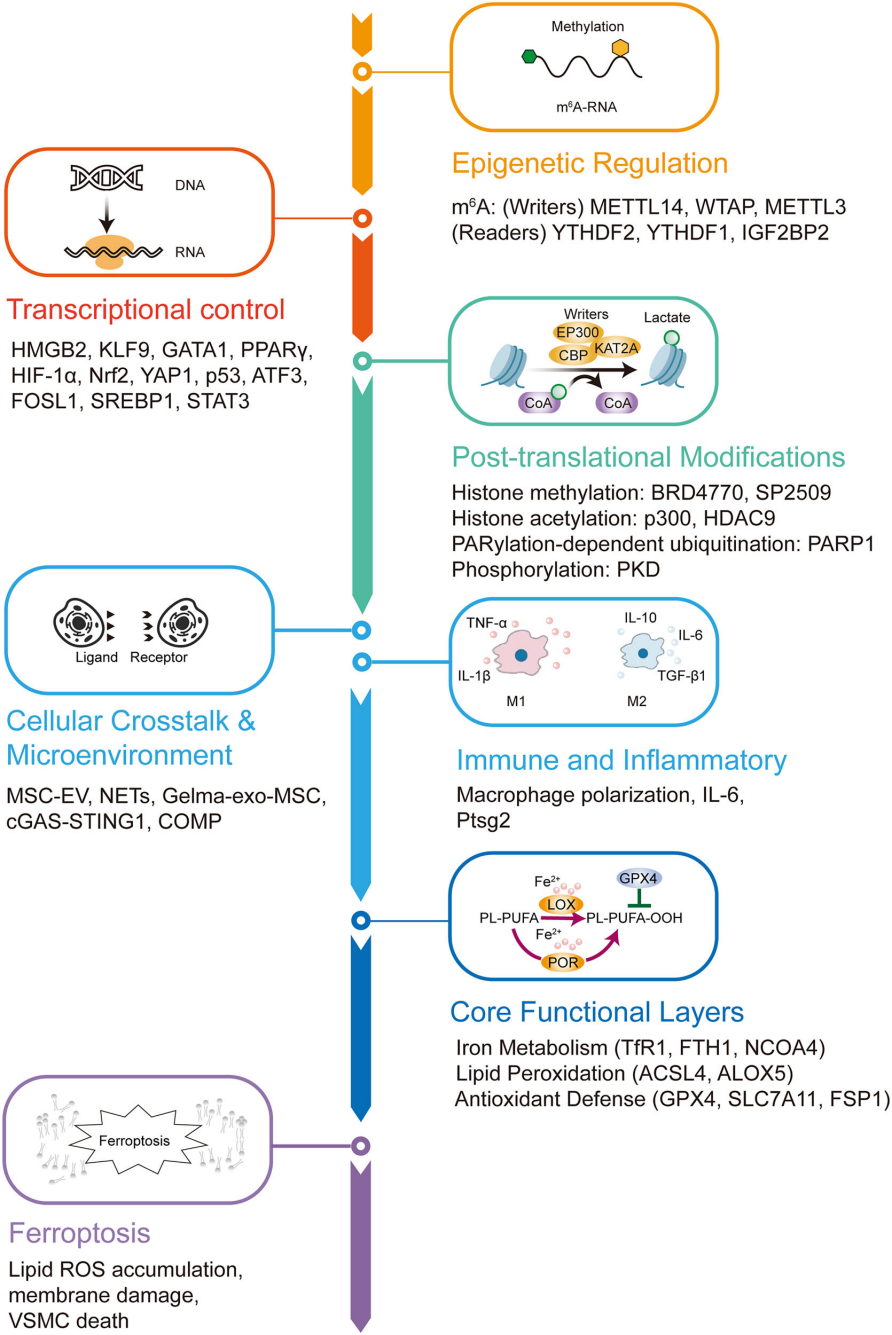
Integrated regulatory network of ferroptosis in vascular smooth muscle cells (VSMCs). Upstream regulators, including epigenetic, transcriptional, and post-translational mechanisms, cooperate with extracellular and immune signals to modulate ferroptosis in vascular smooth muscle cells (VSMCs). These pathways converge on three functional modules: iron metabolism, lipid peroxidation, and antioxidant defense, which culminate in ferroptotic membrane damage and VSMC death.

Ferroptosis in VSMCs is intricately associated with multiple cellular processes, including cell death, senescence, and proliferation. Based on the underlying pathological mechanisms, the role of VSMC ferrptosis can be categorized into three major aspects across different vascular diseases. These distinct yet interconnected functions highlight the complex involvement of ferroptosis in the pathogenesis of diverse vascular pathologies. Moreover, ferroptosis in VSMCs is governed by multi-level regulatory mechanisms. Epigenetic regulation, such as N^6^-methyladenosine (m^6^A) RNA methylation, key enzymes, including METTL14, WTAP, METTL3, modulate the stability of key ferroptosis-related mRNAs including *SLC7A11*, *FSP1*, and *ASCL4*. At the transcriptional level, factors like Nrf2, p53, and HIF-1α coordinate antioxidant defense and lipid metabolism to determine ferroptotic sensitivity. Post-translational modifications (e.g., methylation, acetylation, ubiquitination or phosphorylation of key proteins) further regulate enzymatic activity and protein stability. Intercellular cross-talk within the vascular microenvironment, including, NETs, MSC-EV, Gelma-exo-MSC, cGAS-STING1, and COMP, further modulate iron metabolism and lipid peroxidation. In addition, immune and inflammatory responses have emerged as key regulatory layers in this process. Collectively, these mechanisms complement the epigenetic and molecular regulatory network, providing a more integrated understanding of ferroptosis in VSMCs as depicted in Fig. 2.

### VSMC ferroptosis and cell death in vascular diseases

Ferroptosis-induced VSMC death plays a pivotal role in the pathogenesis of vascular diseases such as AAs, aortic dissection, and IAs. This highlights the detrimental role of ferroptosis, where excessive or uncontrolled ferroptotic injury compromises vascular wall integrity, promoting aneurysm progression, dissection, or rupture.

#### Aortic aneurysm

AA is characterized by the weakening of the vessel wall and progressive aortic lumen dilation, which may remain asymptomatic until dissection or rupture occurs, events that are often fatal [37]. Studies have demonstrated that impaired VSMC function is one of the key mechanisms underlying the pathogenesis of AAs [38]. We organize this article about AA according to the relevant mechanisms.

##### Epigenetic regulation

N^6^-methyladenosine (m^6^A) modification of RNA is a form of post-transcriptional methylation widely present in eukaryotic cells, and it plays a pivotal role in the development and progression of various cardiovascular diseases [39, 40]. Wang et al. demonstrated that methyltransferase 14, N^6^-adenosine-methyltransferase non-catalytic subunit (METTL14) induces ferroptosis in VSMC during the progression of TAA by catalyzing the m^6^A modification of ACSL4 mRNA. Specifically, IGF2BP2 recognizes METTL14-mediated m^6^A-modified ACSL4 mRNA and enhances its stability, thereby promoting ferroptosis in VSMC [41].

Tian et al. found that AngII stimulation enhances the expression of Wilms’ tumor 1-associated protein (WTAP), which mediates m^6^A modification and stabilizes the mRNA of brain abundant membrane attachment signal protein 1 (BASP1). As a result, BASP1 mRNA and protein levels are upregulated, leading to increased ferroptosis in VSMCs and contributing to the development of abdominal aortic aneurysm (AAA) [42]. These findings highlight the WTAP/BASP1 axis as a potential therapeutic target for the treatment of AAA.

##### Transcriptional control

High mobility group box (HMGB) proteins are DNA-binding proteins that regulate DNA transcription, replication, and repair processes [43]. Wu et al. found that the inhibition of HMGB2-regulated VSMC ferroptosis and inflammation, as well as the prevention of AAA via NF-κβ signaling, suggests that HMGB2 could serve as an effective therapeutic target for AAA [44].

##### Cellular cross-talk and microenvironment

Mesenchymal stem cells (MSCs) showed promising therapeutic potential in preventing the development of AAAs by reducing the degradation of elastic fibers in the aortic wall [45]. Chen et al. discovered that extracellular vesicles derived from MSCs (MSC-EVs) inhibit neutrophil extracellular trap (NET) formation, activate the PI3K/AKT signaling pathway, and prevent ferroptosis in VSMCs, thereby attenuating AAA progression [46]. This study identified several potential molecular targets for the development of novel pharmacological approaches and suggested that MSC-EVs could serve as a promising therapeutic strategy for AAA.

Qi et al. demonstrated that NETs were found to modulate the stability of the mitochondrial carrier protein solute carrier family 25- member 11 (SLC25A11), resulting in reduced levels of mitochondrial glutathione (mitoGSH) and elevated mitochondrial malondialdehyde (mitoMDA) production in VSMCs. The consequent depletion of mitoGSH leads to excessive ROS accumulation and triggers ferroptosis in VSMCs, thereby contributing to the development of AAA [47]. These findings suggest that preventing mitoGSH depletion and inhibiting ferroptosis in VSMCs may offer promising therapeutic targets for AAA treatment.

##### Immune and inflammatory

Eugenol, a phenylpropanoid phenolic compound extracted from clove essential oil, is known for its antibacterial and anti-inflammatory properties [48]. Huang et al. demonstrated that eugenol inhibits AngII-induced VSMC death, inflammation, and ferroptosis by targeting the STAT3/HMGB2 signaling axis [49]. Through this mechanism, eugenol effectively attenuates the progression of AAA, highlighting its potential as a therapeutic agent in vascular disease.

##### Core functional layers of ferroptosis

He et al. discovered that Fer-1 may inhibit lipid peroxidation and ferroptosis in mouse aortic vascular smooth muscle cells (MOVAS) by activating the SLC7A11/GPX4 signaling pathway, thereby ameliorating structural abnormalities of the vascular wall in mice with AAA [50]. This study highlights the role of Fer-1 in AAA formation and its associated molecular mechanisms, providing a novel research direction for the clinical treatment of AAA.

Zhou et al. demonstrated that AngII induces ferroptosis in VSMC, while Fer-1, a potent ferroptosis inhibitor, markedly counteracts this effect. Mechanistically, Fer-1 preserves the contractile phenotype of VSMCs, suppresses inflammatory responses, and reduces ECM remodeling [51].

The study by Shi et al. revealed that overexpression of GPX4 inhibits IL-6-induced activation of the JAK1/STAT3 signaling pathway in VSMCs, thereby reducing the recruitment and polarization of pro-inflammatory macrophages. Moreover, GPX4 overexpression effectively suppresses ferroptosis and delays the progression of AAA [52]. These findings underscore the critical role of ferroptosis in AAA pathogenesis and suggest that targeting GPX4 may represent a promising therapeutic strategy for the clinical management of AAA.

Smoking is a major risk factor for AA. However, the mechanisms through which smoking contributes to the development of AA remain incompletely understood. Loss of VSMCs within the medial layer of the arterial wall represents a key pathophysiological hallmark of AA, which can result in the weakening of the vessel wall and subsequent dilatation [53, 54]. Sampilvanjil et al. reported that cigarette smoke extract induces upregulation of prostaglandin-endoperoxide synthase 2 mRNA, lipid peroxidation, and depletion of intracellular GSH, all of which are the hallmark features of ferroptosis. The study further demonstrated that ferroptosis mediates cigarette smoke extract-induced VSMC death, identifying ferroptosis as a potential therapeutic target for the prevention of AA [55].

He et al. demonstrated that phenylthio-modified dendritic polylysine loaded with selenomethionine effectively reduces intracellular ROS levels, upregulated GPX4 expression, inhibits lipid peroxidation, and consequently attenuates the progression of AAA [56]. These findings suggest that phenylthio-modified dendritic polylysine loaded with selenomethionine may serve as a promising nanotherapeutic strategy for the treatment of AAA by targeting ferroptosis.

Schoenmakers et al. showed that oxidative stress-mediated aortic cell ferroptosis predisposed to thoracic aortic aneurysm (TAA) formation in humans and animal models with selenoprotein deficiency. Researchers observed that aortas from patients and animal models exhibit increased cellular ROS levels, oxidative DNA damage, and VSMC apoptosis. Treatment with antioxidants or iron chelators prevented oxidative injury in patient-derived cells and reduced aortic lesions in zebrafish models [57]. Although this study suggests a ferroptosis-dependent effect, alternative forms of oxidative cell death cannot be completely excluded.

He et al. revealed that overexpression of 5-aminolevulinate synthase 2 (ALAS2) is found to mitigate oxidative stress induced by H₂O₂ and suppress iron-dependent ferroptosis in MOVAS cells. However, silencing of GATA-binding protein 1 (GATA1) partially offset this protective effect. These results imply that the GATA1-ALAS21 regulatory axis may serve as a promising therapeutic target for the treatment of AAs [58].

##### Others

Circular RNAs (circRNAs) play critical regulatory roles in the pathogenesis of TAA [59]. Fu et al. reported that knockdown of circCDYL (circ_0008285) mitigates AngII-induced VSMC apoptosis, ferroptosis, and oxidative stress via miR-1270/ a disintegrin and metalloproteinase 10 pathway in TAA [60].

Several important genes and proteins also play critical roles in ferroptosis. GM3, a sialic acid-containing glycosphingolipid (ganglioside), is predominantly present in the liver, muscle, and plasma of both humans and mice [61]. It is synthesized through the addition of sialic acid to lactosylceramide, a reaction catalyzed by ST3GAL5 (GM3 synthase). Zhang et al. demonstrated that GM3 can bind to transferrin receptor 1 (TFR1) on the cell membrane, thereby inhibiting iron uptake and offering new insights into the regulation of VSMC ferroptosis in abdominal AAA. Furthermore, the transcription factor KLF9 suppresses GM3 biosynthesis by inhibiting ST3GAL5 expression through direct binding to its proximal promoter and distal enhancer [62]. These findings suggest that GM3 supplementation could represent a potential therapeutic strategy for AAA.

Sun et al. found that inhibiting ferroptosis-associated signaling pathways facilitated the nuclear-cytoplasmic translocation of peroxisome proliferator-activated receptor gamma (PPARγ), disrupted autophagy centered on the ferroptosis-related ferritin complex involving nuclear receptor coactivators, and consequently attenuated vascular aging-like phenotypes both in vitro and in vivo [63]. These effects conferred protection against vascular ageing-associated diseases such as AAA.

These results underscore the pivotal involvement of VSMC ferroptosis in the development of AA and indicated that targeting ferroptosis could effectively mitigate pathological vascular remodeling, providing a promising therapeutic approach for AA prevention. Table 1 summarizes the underlying mechanisms of therapeutic strategies targeting VSMC ferroptosis in AA.

**Table 1:** Ferroptosis of VSMCs in aortic aneurysm.

Category	Vascular disease	Cells	Treatments	Mechanism
Cardiovascular diseases	aortic aneurysm	HASMC	AngII liproxstatin-1	AngII→METTL14↑→ACSL4 mRNA m^6^A modification↑→IGF2BP2 recognize→ACSL4 mRNA stability↑, translation↑→ACSL4↑→VSMC ferroptosis↑→TAA↑ [41]
	aortic aneurysm	HASMC	AngII	WTAP→BASP1 m^6^A modification↑→BASP1↑→VSMC ferroptosis↑→AAA↑ [42]
	aortic aneurysm	VSMC	AngII erastin	HMGB2 deficiency + AngII→NF-κβ pathway↓→VSMC ferroptosis and inflammation↓→AAA↓ [44]
	aortic aneurysm	HASMC	AngII 740 Y-P Fer-1 Padi4 deficiency	MSC-EV→NET↓→PI3K/AKT↑→VSMCferroptosis↓→AAA↓ [46]
	aortic aneurysm	HASMC	AngII Fer-1 Padi4 KO	NETs→SLC25A11↓→mito GSH↓, mito MDA↑, ROS↑→VSMC ferroptosis↑→AAA↑ [47]
	aortic aneurysm	HASMC	AngII	Eugenol→STAT3/HMGB2↓→VSMC ferroptosis↓, cell proliferation↑, cell death↓, inflammation↓→AAA↓ [49]
	aortic aneurysm	MOVAS	AngII Fer-1 RSL3	AngII+Fer-1→SLC7A11, GPX4↑→VSMC ferroptosis↓→AAA↓ [50]
	aortic aneurysm	MOVAS	AngII Fer-1 Erastin	Fer-1+AngII→VSMC ferroptosis↓→VSMC contractile function↑, inflammation↓, vascular remodeling↓→AAA↓ [51]
	aortic aneurysm	MOVAS	AngII	GPX4↑→IL-6↓→JAK1/STAT3↓→pro-inflammatory macrophages↓→ferroptosis↓→AAA↓ [52]
	aortic aneurysm	rat VSMC	CSE Fer-1 liproxstatin-1	CSE→Ptgs2 mRNA↑→lipid peroxidation↑, GSH↓→VSMC ferroptosis→VSMC loss→AA [55]
	aortic aneurysm	VSMC	AngII	PDPs-Se→ROS↓, GPX4↑→lipid peroxidation↓→VSMC ferroptosis↓→AAA↓ [56]
	aortic aneurysm	HASMC MOVAS	AngII α-tocopherol MitoQ Desferrioxamine	AngII+Secisbp2 deficiency→ROS↑, oxidative DNA damage↑, VSMC apoptosis↑→ferroptosis→aortic aneurysm [57]
	aortic aneurysm	MOVAS	H_2_O_2_	GATA1→ALAS2↑→oxidative stress↓→VSMC ferroptosis↓→TAA↓ [58]
	aortic aneurysm	VSMC	AngII	Knockdown of circCDYL+AngII→miR1270/ADAM10↓→VSMC apoptosis↓, ferroptosis↓,oxidative stress↓→TAA↓ [60]
	aortic aneurysm	HASMC	AngII GM3	KLF9→ST3GAL5 deficiency→GM3↓→transferrin and TFR1 interaction↑→iron accumulation↑→HASMC ferroptosis↑→AAA↑ [62]
	aortic aneurysm	human carotid VSMC	AngII plus bleomycin	PPARγ→NCOA4→ferritinophagy→ferroptosis↑→P16↑, P21↑, NAD^+^↓→senescence↑→AA↑ [63]

↑indicates elevation or activation; ↓indicates reduction or suppression. *CSE* cystathionine γ lyase, *Ptgs2* prostaglandin-endoperoxide synthase 2, *GSH* glutathione, *AA* aortic aneurysm, *KLF9* KLF transcription factor 9, *ST3GAL5* ST3 beta-galactoside alpha-23-sialyltransferase 5, *TFR1* transferrin receptor 1, *HASMC* human aorta vascular smooth muscle cells, *AAA* abdominal aortic aneurysm, *MOVAS* mouse aortic vascular smooth muscle cells, *Secisbp2* SECIS binding protein 2, *MSC-EVs* mesenchymal stem cell-derived extracellular vesicles, *NETs* neutrophil extracellular traps, *PI3K* phosphatidylinositol-45-bisphosphate 3-kinase catalytic subunit beta, *AKT* AKT serine/threonine kinase 1, *HMGB2* high mobility group box 2, *NF-κB* nuclear factor kappa-B, *SLC7A11* solute carrier family 7 member 11, *GPX4* glutathione peroxidase 4, *ADAM10* a disintegrin and metalloproteinase 10, *SLC25A11* solute carrier family 25 member 11, *GATA1* GATA-binding protein 1, *ALAS2* 5-aminolevulinate synthase 2, *TAA* thoracic aortic aneurysm, *PPARγ* peroxisome proliferator-activated receptor gamma, *P16* cyclin dependent kinase inhibitor 2A, *P21* cyclin dependent kinase inhibitor 1A, *METTL14* methyltransferase 14 N6-adenosine-methyltransferase non-catalytic subunit, *WTAP* Wilms’ tumor 1-associated protein, *BASP1* brain abundant membrane attached signal protein 1, *PDPs-Se* phenylthio-modified dendritic polylysine loaded with selenomethionine, *IL-6* interleukin 6, *JAK1* Janus kinase 1, *STAT3* signal transducer and activator of transcription 3.

#### Aortic dissection

Acute Stanford type A aortic dissection (ATAAD) is marked by a tear in the intima and the rapid formation of a false lumen. As the false lumen expands, it can lead to severe complications such as aortic valve insufficiency, pericardial tamponade, or even rupture of the aorta. Compared with type B dissections, ATAAD carries a significantly higher risk of mortality and typically necessitates urgent surgical intervention [64]. Injury and death of VSMCs are thought to contribute substantially to the pathological microenvironment within the false lumen [65]. We elaborate on the articles about aortic dissection, respectively, according to the relevant mechanisms.

##### Epigentic regulation

Li et al. revealed that the expression of the key m^6^A RNA methyltransferase METTL3 is significantly elevated in patients with thoracic AA and dissection (TAAD). Notably, METTL3 protein levels negatively correlated with SLC7A11 and FSP1 expression in human aortic tissue, thereby facilitating VSMC ferroptosis [66]. These findings suggest that targeting ferroptosis or METTL3-mediated m^6^A RNA methylation may represent a promising therapeutic strategy for AD. However, epigenetic regulators provide promising intervention points; their off-target effects and reversibility require further clarification.

Non-coding RNAs activated by DNA damage (NORAD) are lncRNAs that play a crucial role in preserving genomic stability [67]. Liao et al. showed that methyltransferase 3, N^6^-adenosine-methyltransferase complex catalytic subunit (METTL3)-mediated m^6^A modification of NORAD regulates its abundance in a YTHDF2-dependent manner, suppresses VSMC ferroptosis, and mitigates the progression of aortic aneurysm and dissection (AAD) through the ELAV-like RNA binding protein 1 (HUR)/GPX4 signaling axis [68]. These findings indicate that NORAD could serve as a potential therapeutic target for aortic dissection. Although several studies implicate METTL3-mediated m^6^A modification in ferroptosis regulation, its directionality appears cell-type-specific, highlighting the need for single-cell and spatial studies to delineate ferroptosis heterogeneity.

##### Transcriptional control

Heme oxygenase-1 (HO-1) is a critical enzyme responsible for degrading heme, a process that facilitates iron accumulation and consequently promotes ferroptosis [69]. Song et al. [70] revealed that oxidative stress induces VSMC ferroptosis via activation of the HIF-1α/HO-1 pathway, thereby contributing to the development of ATAAD.

Feng et al. reported that lactate dehydrogenase a (LDHA) serves as a novel promoter of ferroptosis in VSMCs during AD. Elevated LDHA expression suppresses the levels of GPX4, SLC7A11, and FSP1. Moreover, an interaction between LDHA and nuclear factor erythroid 2-related factor 2 (NRF2) has been identified. Overexpression of NRF2 or treatment with its agonist effectively counteracted the effects of LDHA, thereby mitigating ferroptosis and lipid peroxidation in VSMCs [71]. These findings identified LDHA as a promising therapeutic target for AD, suggesting that suppressing its expression could offer a novel intervention strategy.

##### Post-translational modifications

Chen et al. demonstrated that the histone methyltransferase inhibitor BRD4770 protects VSMCs from ferroptosis and prevents the development of aortic dissection (AD). Mechanistically, BRD4770 inhibits mono-, di-, and tri-methylation of histone H3 at lysine 9 (H3K9me1/2/3), thereby reactivating key ferroptosis defense pathways, including the System Xc⁻-GPX4 axis, the FSP1-CoQ10 axis, and the GTP cyclohydrolase 1 (GCH1)-BH4 pathway. Moreover, BRD4770 also attenuates the progression of AD by suppressing the inflammatory response [72]. Elucidating the anti-ferroptotic effects of BRD4770 may offer valuable insights into developing therapeutic strategies targeting VSMC ferroptosis in AD.

E1A-associated 300-kDa protein (P300), a well-known histone acetyltransferase, has been implicated in various cardiovascular diseases [73, 74]. Research by Shi et al. demonstrated that loss of P300 function activates the HIF-1α/HMOX1 signaling axis, leading to enhanced lipid peroxidation and promoting ferroptosis in VSMCs [75]. These findings highlight P300 as a promising target for AD intervention.

Histone methylation has been implicated in the pathogenesis of AD [76]. SP2509, a selective inhibitor of lysine-specific demethylase 1 (LSD1) [77], modulates numerous biological processes [78, 79]. He et al. illustrated that SP2509 upregulates the expression of H3K4me2, inhibits the transferrin receptor (TFR) and ferritin expression. Therefore, intracellular iron levels in VSMC are reduced. This could effectively alleviate lipid peroxidation and VSMC ferroptosis in AD [80]. These results indicate that SP2509 hold potential as a therapeutic agent for mitigating AD by decreasing iron accumulation and inhibiting ferroptosis in VSMC.

##### Cellular cross-talk and microenvironment

Wang et al. showed exosomes originating from mesenchymal stem cells (exo-MSCs) represent a promising approach to reverse medial degeneration of the aorta and counteract ferroptosis during AD. In this study, exo-MSCs are incorporated into Gelma hydrogels (Gelma-exos) through ultraviolet crosslinking, combined with three-dimensional (3D) printing technology. The sustained release of exosomes prevents the phenotypic transition of VSMC to a proliferative state, thereby suppressing their proliferation and migration. Moreover, the 3D-printed Gelma-exos effectively inhibited ferroptosis in vitro, in vivo, and in ex vivo experiments [36]. This study demonstrated that Gelma-exos, integrated with 3D printing technology, provide a novel therapeutic strategy for repairing aortic medial degeneration and mitigating ferroptosis in AD, which could potentially alleviate the risk of AD rupture.

##### Others

Tankyrase 1 (TNKS1), a key member of the poly(ADP-ribose) polymerase (PARP) family, plays an essential role in regulating growth and homeostasis [81]. Xu et al. identified TNKS1 as a pivotal regulator in the pathogenesis of AD. Specifically, TNKS1 overexpression promotes ferroptosis and induces phenotypic switching of HASMCs from a contractile to a synthetic state, thereby contributing to aortic wall instability and dissection [82]. These findings highlight TNKS1 and the ferroptosis pathway as promising therapeutic targets for the prevention and treatment of AD.

Table 2 provides a comprehensive summary of therapeutic interventions and mechanistic insights related to VSMC ferroptosis in AD.

**Table 2:** Ferroptosis of VSMCs in aortic dissection.

Category	Vascular disease	Cells	Treatments	Mechanism
Cardiovascular Diseases	aortic dissection	HASMC	AngII	Gelma-exo-MSC→VSMC phenotype switching↓, proliferation and migration↓, GPX4↑, lipid ROS↓→VSMC ferroptosis↓→ AD↓ [36]
	aortic dissection	HASMC	IKE CD	METTL3→SLC7A11↓, GPX4↓, FSP1↓→VSMC ferroptosis↑→AD↑ [66]
	aortic dissection	MOVAS	AngII	AngII→METTL3↑→NORAD m^6^A methylation↑→YTHDF2 recognize→NORAD↓→HUR/GPX4↓→VSMC ferroptosis↑→AD↑ [68]
	aortic dissection	HASMC	H_2_O_2_	Oxidative stress→HIF1α/HO-1↑→iron↑→VSMC ferroptosis↑→AD↑ [70]
	aortic dissection	HASMC	CD IKE	LDHA→NRF2↓→FSP1↓, GPX4↓, SLC7A11↓→VSMC ferroptosis↑→AD↑ [71]
	aortic dissection	HASMC	CD IKE RSL3 BRD4770	BRD4770→H3K9me1/2/3↓→SLC7A11↑, GPX4↑, FSP1↑, GCH1↑→lipid peroxidation↓→VSMC ferroptosis↓→inflammatory response↓→AD↓ [72]
	aortic dissection	HASMC	CD IKE Fer-1	P300 deficiency→HIF-1α/HMOX1↑→lipid peroxidation↑→VSMC ferroptosis↑→AD↑ [75]
	aortic dissection	HASMC	CD IKE SP2509 LPS	SP2509→H3K4me2↑→TFR↓→Fe^3+^↓→Fe^2+^↓→fenton reaction↓, inflammation↓→lipid peroxidation↓→VSMC ferroptosis↓→AD↓ [80]
	aortic dissection	HASMC	BAPN	TNKS1↑→ferroptosis↑→HASMC phenotypic switch↑→AD↑ [82]

↑indicates elevation or activation, ↓indicates reduction or suppression. *HASMC* human aorta vascular smooth muscle cells, *CD* cystine deficiency, *IKE* imidazole ketone erastin, *FSP1* ferroptosis suppressor protein 1, *GCH1* GTP cyclohydrolase 1, *AD* aortic dissection, *METTL3* methyltransferase 3 N6-adenosine-methyltransferase complex catalytic subunit, *SLC7A11* solute carrier family 7 member 11, *GPX4* glutathione peroxidase 4, *P300* E1A-associated 300-kDa protein, *HIF-1α* hypoxia-inducible factor-1α, *HO-1* heme oxygenase-1, *HMOX1* heme oxygenase 1, *NORAD* non-coding RNA activated by DNA damage, *YTHDF2* YTH N6-methyladenosine RNA binding protein F2, *HUR* ELAV like RNA binding protein 1, *LPS*, lipopolysaccharides, *TFR* transferrin receptor, *Gelma-exo-MSC* exosomes derived from mesenchymal stem cells-embedded in Gelma hydrogels, *LDHA* lactate dehydrogenase A, *NRF2* nuclear factor (erythroid-derived 2)-like 2, *BAPN* β-aminopropionitrile, *TNKS1* Tankyrase 1.

#### Intracranial aneurysm

Intracranial aneurysm (IA) is a pathological dilation of a major cerebral artery branch affecting ~3–5% of adults worldwide [83]. A hallmark pathological feature of human IA is the dysfunction or loss of VSMCs, which are essential for maintaining the contractile function of the vessel wall. The depletion of VSMCs leads to structural defects in the arterial wall, promoting the formation of IAs [84, 85].

Krüppel-like factor 15 (KLF15) is recognized as a vascular protective factor that plays a crucial role in maintaining vascular homeostasis [86–88]. Fang et al. demonstrated that KLF15 reduced the sensitivity of VSMCs to ferroptosis by interacting with p53, thereby preventing p53-mediated repression of SLC7A11 transcription [89]. These findings highlight the protective role of KLF15 in shielding Human brain vascular smooth muscle cells from ROS-induced ferroptosis.

### VSMC ferroptosis and cell senescence in vascular diseases

Ferroptosis may also contribute to VSMC senescence, a process implicated in the development of atherosclerosis and vascular calcification. Mechanistically, iron accumulation and lipid peroxidation can promote oxidative stress and DNA damage, thereby accelerating cellular aging and dysfunction.

#### Atherosclerosis

Atherosclerosis (AS) is the vital pathological basis of cardiovascular diseases (CVD) and remains the leading cause of morbidity and mortality of CVD around the world [90]. Studies have shown that fragile plaques are characterized by a thin, fragile fibrous cap, primarily composed of VSMC, reduced collagen fibers content, significant infiltration of macrophages and lymphocytes, and large necrotic cores containing numerous cholesterol crystals [91–93]. We discuss studies on AS according to their underlying mechanisms.

##### Transcription control

Zhang et al. showed that echinatin, which is a chalcone predominantly found in roots and rhizomes of licorice, reduces the stiffness of ECM surrounding cultured VSMCs. Mechanistically, echinatin activates nuclear factor, erythroid-derived 2, like 2 (Nrf2)/glutamate-cysteine ligase catalytic subunit (GCLC)/glutamate-cysteine ligase, modifier subunit (GCLM) signaling axis, which maintains the GSH homeostasis and thereby inhibits ferroptosis. Echinatin further downregulated the expression of collagen type I alpha 1 chain (Col1a1) and collagen type III alpha 1 chain (Col3a1), alleviating ECM remodeling and progression of AS [94]. This finding, while compelling, is derived mainly from rodent models and may not fully reflect human vascular physiology.

You et al. reported that inhibition of ferroptosis in VSMCs reduces AS lesions in mice fed a high-fat diet (HFD). They also demonstrated that Ferrostatin-1 (Fer-1) inhibits HFD-induced ferroptosis in aortic VSMCs. Mechanistically, the NRF2/FSP1 axis is the key endogenous antioxidant pathway mediating the inhibition of VSMC ferroptosis and AS, although the precise regulatory mechanisms remain fully elucidated [95]. Further investigation into the relationship between VSMC ferroptosis and AS may provide novel therapeutic strategies and targets for atherosclerotic cardiovascular disease and its clinical complications.

##### Core functional layers of ferroptosis

Guo et al. reported that catechin, a promising antioxidant, abundantly found in green tea, effectively reduces oxidative stress and alleviate ox-LDL-induced VMSC ferroptosis through activation of the Nrf2/solute carrier family 7 member 11 (SLC7A11)/GPX4 signaling axis, indicating its positive function in preventing atherosclerosis [96].

##### Others

Chen et al. identified Yes-associated protein 1 (YAP1) as an anti-ferroptosis regulator, that promotes the expression of glutaminase 1 (GLS1). GLS1 upregulates the expression of glutamate (Glu) and GSH, thereby inhibiting VSMC ferroptosis and contributing to the stabilization of atherosclerotic plaques [97].

Li et al. identified that heat shock protein B1 (HSPB1) is a novel regulator in VSMC ferroptosis. This study demonstrated that HSPB1 inhibits oxidized low-density lipoprotein (oxLDL)-induced VSMC ferroptosis by suppressing dipeptidyl peptidase 4 (DPP4) via nuclear factor kappa-B (NF-κB). These findings suggest that the modulation of HSPB1activity could provide new avenues for the prevention and therapy of atherosclerosis [98]. However, this mechanism has primarily been demonstrated in vitro, and its in vivo relevance remains to be confirmed.

The underlying mechanisms of therapeutic interventions and mechanisms targeting VSMC ferroptosis in atherosclerosis are summarized in Table 3.

**Table 3:** Ferroptosis of VSMCs in atherosclerosis.

Category	Vascular disease	Cells	Treatments	Mechanism
Cardiovascular diseases	atherosclerosis	HUASMC	High phosphate Echinatin	Echinatin→Nrf2/GCLC/GCLM→GSH↑→ferroptosis↓→matrix remodeling (Col1a1↑, Col3a1↑) [94]
	atherosclerosis	HASMC MOVAS	oxLDL Fer-1	oxLDL→TFR1↑, NRF2↓, FSP1↓SOD-1↓→VSMC ferroptosis↑→atherosclerosis↑ [95]
	atherosclerosis	MOVAS	oxLDL catechin	Catechin→Nrf2/SLC7A11/GPX4↑→ferroptosis↓→atherosclerosis↓ [96]
	atherosclerosis	MOVAS	oxLDL Fer-1	YAP1→GLS1→Glutamate→GSH↑→ferroptosis↓→atherosclerosis↓ [97]
	atherosclerosis	rat VSMC	oxLDL liproxstain-1 DFO	HSPB1→NF-κB↓→DPP4↓→fe-roptosis↓→atherosclerosis↓ [98]

↑indicates elevation or activation, ↓indicates reduction or suppression. *HUASMC* human umbilical artery smooth muscle cells, *Nrf2* nuclear factor E2-related factor 2, *GCLC* glutamate-cysteine ligase catalytic subunit, *GCLM* glutamate-cysteine ligase modifier subunit, *GSH* glutathione, *Col1a1* collagen type I alpha 1 chain, *Col3a1* collagen type III alpha 1 chain, *MOVAS* mouse aortic vascular smooth muscle cells, *oxLDL* oxidized low-density lipoprotein, *HASMC* human aorta vascular smooth muscle cells, *Fer-1* ferrostatin-1, *FR1* transferrin receptor 1, *FSP1* ferroptosis suppressor protein 1, *SOD1* superoxide dismutase 1, *YAP1* Yes-associated protein 1, *GLS1* glutaminase 1, *HSPB1* heat shock protein B1, *NF-κB*, nuclear factor kappa-B, *DPP4* dipeptidyl peptidase 4, *SLC7A11* solute carrier family 7 member 11, *GPX4* glutathione peroxidase 4.

#### Vascular calcification

Vascular calcification is commonly observed in patients with chronic kidney disease (CKD), diabetes, and atherosclerosis [99–101]. It is characterized by abnormal calcium phosphorus crystal deposition within the vascular wall [102]. Recent studies have demonstrated that vascular calcification as an active and tightly regulated pathological process, involving VSMC phenotypic transformation, resembling osteogenesis [92, 103, 104]. We summarize the studies on vascular calcification based on the distinct molecular and cellular mechanisms involved.

##### Transcription control

Metformin, a commonly prescribed biguanide antihyperglycemic drug, has also been shown to confer multiple cardiovascular protective effects [105, 106]. Ma et al. found that metformin inhibits the palmitic acid (PA)-induced upregulation of periostin (POSTN), which in turn increases p53 expression in VSMCs. This promotes the expression of SLC7A11 and GPX4, suppresses lipid peroxidation, and alleviates ferroptosis in VSMCs, ultimately reducing vascular calcification. Additionally, metformin enhances the antioxidant capacity of VSMCs by activating the nuclear factor E2-related factor 2 (Nrf2) signaling pathway [107]. Collectively, these findings suggest that targeting POSTN in VSMCs may offer a promising therapeutic strategy for the prevention and treatment of vascular calcification. Although metformin has shown anti-ferroptotic effects, whether this benefit is direct or secondary to systemic metabolic improvement remains unclear.

Fos-like antigen 1 (FOSL1) is a transcription factor whose activity is regulated at multiple levels, including transcriptional and post-translational modifications [108]. Previous studies have demonstrated that FOSL1 participates in various cellular processes, especially those related to inflammation and cell death [109]. Notably, Shao et al. reported that FOSL1 inhibits ferroptosis in VSMCs via the p53-SLC7A11 axis, thereby alleviating vascular calcification and reducing ROS production [110]. These findings suggest that downregulation of FOSL1 may represent a potential therapeutic strategy for the prevention of vascular calcification.

Coronary artery calcification (CAC) is a hallmark of vascular degeneration characterized by calcium deposition within the coronary artery walls [111]. Zou et al. demonstrated that overexpression of phosphoglycerate dehydrogenase (PHGDH) attenuated ferroptosis by modulating the p53/SLC7A11 signaling axis, thereby reducing calcification in human coronary artery smooth muscle cells (HCASMCs). This ferroptosis inhibition contributed to the mitigation of vascular calcification [112]. These findings highlight PHGDH as a promising therapeutic target for the prevention and treatment of CAC.

Fisetin (3,3′,4′,7-tetrahydroxyflavone), a naturally occurring flavonoid abundant in various fruits and vegetables, exhibits a broad spectrum of biological activities, including anti-inflammatory, antioxidant, anti-tumor effects [113, 114]. Feng et al. demonstrated that fisetin alleviates vascular calcification by modulating heterogeneous nuclear ribonucleoprotein A1 (HNRNPA1)-mediated ferroptosis in both in vitro and in vivo models [115]. These findings suggest that fisetin may serve as a potential therapeutic agent for vascular calcification through its regulation of ferroptotic pathways.

##### Post-translational modifications

Xiong et al. found that histone deacetylase 9 (HDAC9) promotes ferroptosis and osteogenic trans-differentiation of VSMCs, thereby accelerating vascular calcification in the context of CKD [116]. This study highlights the critical role of HDAC9 in mediating VSMC ferroptosis and offers novel therapeutic insights for the prevention and treatment of vascular calcification in CKD.

Protein kinase D (PKD), a member of the calcium/calmodulin-dependent protein kinase (CAMK) family, has been implicated in vascular pathophysiology [117]. He et al. demonstrated that activation of PKD leads to its autophosphorylation and subsequently promotes ferroptosis by activating the p53-SLC7A11 axis. This signaling cascade facilitates the osteogenic differentiation and calcium deposition of VSMCs, ultimately contributing to the progression of vascular calcification [118]. Post-translational modifications such as phosphorylation may influence ferroptosis signaling, yet definitive mechanistic data are still limited.

Recently, Yang et al. discovered the underlying mechanism of poly[ADP-ribose] polymerase 1 (PARP1)-mediated poly(ADP-ribosyl)ation (PARylation) in VSMC ferroptosis during vascular calcification. Mechanistically, activation of PARP1 in calcified VSMCs elevates POLG PARylation levels, triggering PARylation-dependent ubiquitination and subsequent downregulation of POLG. This downregulation leads to smitochondrial dysfunction and activation of the adenosine receptor A2A (Adora2a)/Ras-associated protein 1 (Rap1) signaling pathway, thereby inducing VSMC ferroptosis and ultimately exacerbating vascular calcification [25]. However, the temporal dynamics of ferroptosis during disease progression remain poorly defined.

##### Core functional layers of ferroptosis

Ye et al. reported that under CKD condition, inhibition of SLC7A11/GSH/GPX4 axis triggers VSMC ferroptosis and promoted vascular calcification, thereby providing a potential therapeutic target for vascular calcification. Specifically, they demonstrated that ferrostatin-1 could alleviate calcification in rat, mice and human atrial rings ex vivo. However, erastin, a small molecule ferroptosis inducer, that depletes GSH, significantly promotes the calcification of VSMCs under osteogenic conditions. The inhibition of RSL3 by GPX4 also promotes VSMCs calcification [35].

1-palmitoyl-2-(5′-oxo-valeroyl)-sn-glycero-3-phosphocholine, an oxidized phospholipid present in atherosclerotic plaques, is known to induce oxidative stress. However, its effect on the osteogenic differentiation and calcification of VSMCs remains unexplored. Lu et al. demonstrated that 1-palmitoyl-2-(5′-oxo-valeroyl)-sn-glycero-3-phosphocholine elevates oxidative stress and disrupts mitochondrial function in VSMCs, thereby promoting lipid peroxidation and inducing ferroptosis in VSMCs, ultimately contributing to vascular calcification. Notably, inhibition of ferroptosis could prevent 1-palmitoyl-2-(5′-oxo-valeroyl)-sn-glycero-3-phosphocholine-induced vascular calcification [119].

Aierken et al. found that the expression of metal ion transporters solute carrier family 39 member 14 (Slc39a14) and solute carrier family 39 member 8 (Slc39a8) is markedly upregulated during vascular calcification. Suppression of these transporters effectively attenuated vascular calcification by preventing iron overload-induced ferroptosis in VSMCs, offering novel therapeutic insights for the management of vascular calcification [120].

Wang et al. reported that lipocalin-2 (LCN2), through the regulation of NCOA4/FTH1 pathway, induces ferritinophagy associated-ferroptosis in VSMCs, thereby promoting vascular calcification in CKD [121]. These findings suggest that targeting LCN2 and VSMC ferroptosis may represent a promising therapeutic approach for the treatment of vascular calcification.

##### Others

Chen et al. illustrated that oleoylethanolamide, an endogenous bioactive mediator involved in lipid homeostasis [122], exerts vascular protective effects against medial calcification by inhibiting VSMC ferroptosis. This effect is attributed to the activation of PPARα and the consequent suppression of ferritinophagy-induced mitochondrial damage via the cGAS–STING1 signaling axis [123]. While this data provides valuable mechanistic insight, extrapolation to human vascular systems should be made with caution.

Wu et al. demonstrated that calcification stimuli significantly downregulate the expression of sterol regulatory element-binding protein 1 (SREBP1). Suppression of SREBP1 expression reduces its binding to the promoter region of inhibitor of apoptosis-stimulating protein of p53 (iASPP), leading to a marked decrease in iASPP levels. This downregulation promotes lipid peroxidation, thereby triggering ferroptosis and exacerbating vascular calcification [124]. These findings suggest that targeting the SREBP1/iASPP axis may represent a promising therapeutic strategy for vascular calcification in CKD.

The underlying mechanisms of therapeutic interventions and mechanisms targeting VSMC ferroptosis in vascular calcification are summarized in Table 4.

**Table 4:** Ferroptosis of VSMCs in vascular calcification.

Category	Vascular disease	Cells	Treatments	Mechanism
Cardiovascular diseases	Vascular calcification	rat VSMC	Ca/Pi Erastin RSL3 Fer-1 NAC	Ca/Pi→ATF3↓→SLC7A11/GSH/GPX4↓→ferroptosis↑→ vascular calcification↑ [35]
	Vascular calcification	rat VSMC	PA/Met	Met→POSTN↓→p53↑→SLC7A11↑, GPX4↑→lipid peroxidation↓→vascular calcification↓; Met→Nrf2→NQO1→lipid peroxidation↓→VSMC ferroptosis↓→vascular calcification [107]
	Vascular calcification	MOVAS	Calcifying media Erastin Fer-1	FOSL1 inhibition→P53/SLC7A11↑→VSMC ferroptosis↓→ROS↓, vascular calcification↓ [110]
	Coronary artery calcification	HCASMC	Ca/Pi Erastin	PHGDH→P53/SLC7A11→ferrptosis↓→CAC↓ [112]
	Vascular calcification	HASMC	High phosphate Fer-1	Fisetin→HNRNPA1→VSMC ferroptosis↓→vascular calcification↓ [115]
	Vascular calcification	A7r5	Ca/Pi	HDAC9→VSMC ferroptosis↑→osteogenic trans-differentiation↑→vascular calcification↑ [116]
	Vascular calcification	MOVAS	Ca/Pi	Ca/Pi→PARP1↑→POLG↓→mitochondrial dysfunction→Adora2a/Rap1↑→SLC7A11/GPX4↓→VSMC ferroptosis↑→vascular calcification↑ [25]
	Vascular calcification	rat VSMC HASMC	POVPC Fer-1	POVPC→mitochondrial dysfuntion→lipid peroxidation↑→ferroptosis↑→vascular calcification↑[119]
	Vascular calcification	MOVAS	Ca/Pi	Slc39a14/Slc39a8↑→FTH1↑→ROS↑→lipid peroxidation↑→VSMC ferroptosis↑→vascular calcification↑ [120]
	Vascular calcification	MOVAS	Ca/Pi	Lipocalin-2→NCOA4/FTH1→ferritinophagy→VSMC ferroptosis↑→vascular calcification↑ [121]
	Vascular calcification	rat VSMC	PA/OEA	PA→PPARα↓→mtDNA damage →cGAS/STING1↑→autophagy-dependent ferrotposis↑→vascular calcification↑ [123]
	Vascular calcification	rat VSMC	Ca/Pi Erastin	SREBP1↓→iASPP↓→lipid peroxidation↑→VSMC ferroptosis↑→vascular calcification↑ [124]

↑indicates elevation or activation; ↓indicates reduction or suppression. *Met* metformin, *POSTN* periostin, *P53* tumor protein p53, *SLC7A11* solute carrier family 7 member 11, *GPX4* glutathione peroxidase 4, *NRF2* nuclear factor E2-related factor 2, *NQO1* NAD(P)H quinone dehydrogenase 1, *Fer-1* ferrostatin-1, *NAC* N-acetylcysteine, *ATF3* activating transcription factor 3, *PA* palmitic acid, *OEA* oleoylethanolamide, *PPARα* peroxisome proliferator activated receptor alpha, *FOSL1* fos-like antigen 1, *POVPC* 1-palmitoyl-2-(5′-oxo-valeroyl)-sn-glycero-3-phosphocholine, *Slc39a14* solute carrier family 39 member 14, *Slc39a8* solute carrier family 39 member 8, *FTH1* ferritin heavy chain 1, *HCASMC* Human coronary artery smooth muscle cell, *PHGDH* phosphoglycerate dehydrogenase, *CAC* coronary artery calcification, *HDAC9* histone deacetylase 9, *NCOA4* nuclear receptor coactivator 4, *HNRNPA1* heterogeneous nuclear ribonucleoprotein A1, *SREBP1* sterol regulatory element-binding protein 1, *iASPP* inhibitor of apoptosis-stimulating protein of p53, *PARP1* poly(ADP-ribose) polymerase 1, *POLG* DNA polymerase gamma catalytic subunit, *Adora2a* adenosine A2a receptor, *Rap1* RAS-related protein 1a.

### VSMC ferroptosis and cell proliferation in vascular diseases

Ferroptosis may paradoxically influence VSMC proliferation, especially under pathological stimuli. In diseases such as hypertension, neointimal hyperplasia, and pulmonary arterial hypertension (PAH), an imbalance between ferroptotic signaling and cell survival pathways may disrupt normal proliferation control, contributing to aberrant vascular remodeling. In these diseases, ferroptosis exhibits a beneficial role: moderate activation can restrain pathological hyperproliferation and neointimal formation.

#### Hypertension

Hypertension is a critical risk factor for ischemic heart disease, stroke, and other cardiovascular disorders [125]. Primary hypertension remains a leading cause of cardiovascular morbidity and mortality worldwide [126]. Therefore, exploring its underlying pathogenesis is of great clinical significance.

Hydrostatic pressure, circumferential stretch, and shear stress are the principal biomechanical forces acting on blood vessels, playing a pivotal role in the onset and progression of hypertension [127]. Jin et al. reported that under high hydrostatic pressure, downregulation of cystathionine γ lyase/hydrogen sulfide (CSE/H_2_S) induce ferroptosis thereby exacerbating VSMC dysfunction. Treatment with inhibitor ferrostatin-1 (Fer-1) effectively suppresses high hydrostatic pressure-induced VSMC inflammation, as evidenced by reduced CXCL2 expression, and prevented endothelial dysfunction-associated phenotype switching marked by decreased AKR1C2 expression. These effects are accompanied by increased GPX4 expression, reduced reactive oxygen species (ROS) levels, and decreased lipid peroxidation. In contrast, the ferroptosis inducer RSL3 enhances high hydrostatic pressure-induced CXCL2 and AKR1C2 expression. Notably, ferroptosis induced by high hydrostatic pressure and RSL3 is attenuated by NaHS administration [128].

#### Neointimal hyperplasia and vascular remodeling

Neointimal hyperplasia following stent implantation remains a key contributor to the failure of percutaneous coronary intervention in the treatment of coronary artery disease [129].

##### Epigentic regulation

Arteriovenous fistula remains the preferred form of vascular access for hemodialysis in patients with end-stage renal disease; however, its long-term patency is frequently compromised by neointimal hyperplasia. Zhao et al. demonstrated that METTL3, a key m^6^A methyltransferase, increases N^6^-methyladenosine (m^6^A) modification of SLC7A11 mRNA, thereby enhancing SLC7A11 stability and translation through recruitment of the m^6^A reader YTH N^6^-methyladenosine RNA binding protein 1 (YTHDF1). This regulatory axis promotes VSMC proliferation, migration, and phenotypic switching, while simultaneously inhibiting ferroptosis, ultimately contributing to vascular intimal hyperplasia in arteriovenous fistula [130]. These findings suggest that targeting the METTL3/SLC7A11/YTHDF1 axis may offer novel therapeutic strategies for improving arteriovenous fistula outcomes.

##### Core functional layers of ferroptosis

The heterocyclic trioxirane compound [1,3,5-tris((oxiran-2-yl)methyl)-1,3,5-triazinane-2,4,6-trione (TGIC)], recognized for its anticancer properties, is used as the core scaffold to conjugate a non-steroidal anti-inflammatory drug, yielding the novel compound BY1 [131]. Zhang et al. reported that OPN-conjugated nanoparticles loaded with BY1 (TOP@MPDA@BY1) effectively target proliferating VSMCs. BY1 promots ferroptosis by enhancing the interaction between NCOA4 and FTH1, leading to increased intracellular ferrous ion levels and subsequent inhibition of vascular restenosis [132]. These findings suggest that BY1 holds strong potential as a candidate for drug-eluting stents or restenosis therapy.

Perfluorooctane sulfonate is a synthetic chemical possessing a long-fluorinated carbon chain, commonly detected in water sources, food products, and their packaging materials [133]. F-53B, a chlorinated alternative to perfluorooctane sulfonate (chemical name: 6:2 chlorinated polyfluorinated ether sulfonate), has been used as a replacement compound. Zhang et al. revealed that F-53B triggers activation of the LOC101929922/miR-542-3p/ACSL4 signaling axis, thereby inducing ferroptosis and phenotypic switching in VSMCs, which contributs to vascular remodeling. Treatment with Fer-1 effectively rescues VSMCs from dysfunction and reverses vascular remodeling induced by F-53B [134]. However, whether ACSL4 upregulation directly drives ferroptosis or reflects secondary lipid metabolic stress requires further experimental confirmation.

Kim et al. demonstrated that Magnolia kobus DC. (MO), a plant-based medicine officially listed in the pharmacopeias of several Asian countries, prevents neointimal hyperplasia by regulating ferroptosis and phenotypic switching of VSMCs in a carotid artery ligation mouse model. Furthermore, MO significantly suppressed ferroptosis-induced cellular dysfunction in erastin-treated A7r5 cells by modulating cell viability, ROS scavenging activity, GSH levels, lipid peroxidation, cell proliferation, and migration [135].

Yu et al. demonstrated that ginger acts as a natural inhibitor of ferroptotic stress. Specifically, 6-gingerol suppresses P53, while 6-shogaol inhibits Alox5 expression. Additionally, both compounds attenuated ferroptotic stress by downregulating PPARγ-dependent Nox1 expression [136]. As a multi-component botanical agent, ginger synergistically combines the protective effects of its constituents to maximize anti-ferroptotic activity, prevent VSMC phenotypic switching, and thereby mitigate vascular remodeling.

Zhang et al. reported a strong association between VSMC ferroptosis and neointima development, characterized by a phenotypic transition of VSMCs from a differentiated contractile state to a dedifferentiated synthetic phenotype. This ferroptosis-driven phenotypic switch is accompanied by increased ROS accumulation [137]. Therefore, these findings suggest that targeting VSMC ferroptosis or suppressing ROS generation may offer effective therapeutic strategies for managing vascular occlusion due to surgical interventions and restenosis post-arterial injury. The studies related to neointimal hyperplasia and vascular remodeling are organized in terms of the specific mechanisms.

##### Others

Mucosa-associated lymphoid tissue lymphoma translocation protein 1 (MALT1) is a human paracaspase possesssing proteolytic activity mediated by its caspase-like domain. Yan et al. reported that MI-2, a selective chemical inhibitor of MALT1, triggers ferroptosis in VSMCs by suppressing mTOR signaling and enhancing autophagy. This leads to reduced contractile function, inhibition of neointimal hyperplasia, and attenuation of atherosclerotic progression [138]. The fact that the study did not analyze the effects of MI-2 on other cell types (e.g., endothelial cells, macrophages), represents the limitation of the current study.

The therapeutic approaches and underlying molecular mechanisms targeting VSMC ferroptosis in neointimal hyperplasia are detailed in Table 5.

**Table 5:** Ferroptosis of VSMCs in neointimal hyperplasia.

Category	Vascular disease	Cells	Treatments	Mechanism
Cardiovascular Diseases	Neointimal hyperplasia	HASMC	PDGF-BB	METTL3→SLC7A11 m^6^A modification↑→YTHDF1→SLC7A11↑→VSMC proliferation↑, migration↑, phenotypic switching↑, ferroptosis↓→neointimal hyperplasia↑ [130]
	Neointimal hyperplasia	MOVAS	BY1	BY1→NCOA4-FTH1 intertaction↑→Fe^2+^↑→VSMC ferroptosis↑→neointimal hyperplasia↓→vascular restenosis↓ [132]
	Vascular remodeling	VSMC	F-53B Fer-1	F-53B→ LOC101929922/miR-542-3p/ACSL4↑→GSH/GPX4/ lipid ROS↑→VSMC ferroptosis↑→VSMC phenotype switch→vascular remodeling↑ [134]
	Neointimal hyperplasia	A7r5	Erastin	MO→cell death↓, ROS↓, cell migration↓→ferroptosis↓→neointimal hyperplasia↓ [135]
	Neointimal hyperplasia	MOVAS	ginger	6-gingerol→Nox1↓, P53↓→ferroptosis↓→neointimal hyperplasia↓; 6-shogaol→Alox5↓, Nox1↓→ferroptosis↓→neointimal hyperplasia↓ [136]
	Neointimal hyperplasia	rat VSMC	RSL3 Fer-1 NAC	vascular injury→ROS↑→ferroptosis↑→VSMC dedifferentiation→neointima formation [137]
	Neointimal hyperplasia	HASMC MOVAS	MI-2 DFO Fer-1	MI-2→MALT1↓→mTOR↓→autography↑→VSMC ferroptosis↑→neointimal hyperplasia↓, AS↓[138]

↑ indicates elevation or activation, ↓ indicates reduction or suppression. *MALT1* mucosa-associated lymphoid tissue lymphoma translocation protein 1, *NCOA4* nuclear receptor coactivator 4, *FTH1* ferritin heavy chain 1, *F-53B* the compound 6:2 chlorinated polyfluorinated ether sulfonate, *ACSL4* acyl-CoA synthetase long-chain family member 4, *METTL3* methyltransferase 3 N6-adenosine-methyltransferase complex catalytic subunit, *YTHDF1* YTH N6-methyladenosine RNA binding protein F1, *MO* Magnolia kobus DC, *Nox1* NADPH oxidase 1, *P53* tumor protein p53, *Alox5*, arachidonate 5-lipoxygenase.

#### Pulmonary arterial hypertension

PAH is a chronic and progressive disease characterized by remodeling of the pulmonary vasculature [139]. Pulmonary vascular remodeling, a hallmark of PAH, is primarily driven by the excessive proliferation and reduced cell death of pulmonary artery smooth muscle cells (PASMCs) [140, 141]. We categorize and discuss the studies on PAH according to mechanisms.

##### Transcription control

Wang et al. uncovered a novel mechanism through which the long non-coding RNA MIR210HG contribute to the progression of hypoxic pulmonary hypertension. Their study revealed that MIR210HG transcription is driven by STAT3, and that MIR210HG enhances intracellular HIF-2α levels by directly interacting with it. This upregulation of HIF-2α subsequently activates autophagy-dependent ferroptosis, facilitating the phenotypic switch of PASMCs from a contractile to a synthetic state under hypoxic conditions [142]. These findings illuminate a previously unrecognized regulatory pathway in HPH pathogenesis and suggest potential molecular targets for therapeutic intervention.

##### Post-translational modifications

Li et al. demonstrated that the chromatin-associated circ RNA SCN8A played a critical role in the regulation of hypoxia-induced ferroptosis in PASMCs. Mechanistically, chromatin-associated circ RNA SCN8A recruites the histone acetyltransferase EP300 lysine acetyltransferase (EP300) to facilitate the lactylation of fused in sarcoma (FUS). This post-translational modification promotes the formation of an R-loop structure between chromatin-associated circRNA SCN8A and the non-host SLC7A11 promoter, thereby regulating the transcriptional activity of SLC7A11 [143]. This regulatory mechanism controls hypoxia-induced ferroptosis in PASMCs.

##### Core functional layers of ferroptosis

Hu et al. reported that SLC7A11 expression is upregulated in both Sugen5416/hypoxia-induced PAH rat models and in patients with PAH. Moreover, overexpression of SLC7A11 in PASMCs suppresses ferroptosis and enhances cell proliferation. Conversely, erastin induced ferroptosis both in vivo and in vitro by downregulating the expression of SLC7A11 and GPX4 [144]. These findings suggest that the aberrant proliferation of hypoxia-induced PASMCs can be attenuated through erastin treatment, thereby revealing potential therapeutic targets and mechanistic insights into the pathogenesis and treatment of PAH.

Liu et al. revealed that the nuclear membrane-localized circular RNA circ-calm4 plays a crucial role in regulating ferroptosis in hypoxia-exposed PASMCs. Mechanistically, circ-calm4 forms a circular RNA-DNA hybrid (circR loop) with the COMP promoter, thereby enhancing COMP transcription. COMP, in turn, modulates the expression of key ferroptosis-related molecules, including TFR1, nicotinamide adenine dinucleotide phosphate oxidase 2 (NOX2), and GPX4, ultimately driving ferroptotic cell death in hypoxic PASMCs [145]. These findings suggest that the circ-calm4/COMP axis may represent a potential therapeutic target for mitigating ferroptosis-associated vascular remodeling and PAH progression.

He et al. indicated that the expression of circMyst4, driven by super-enhancers, is upregulated under hypoxic conditions. The underlying mechanism involved promoting the processing of GPX4 mRNA via DDX5 and inhibiting the interaction between Eef1a1 and ACSL4, thereby suppressing ferroptosis in VSMCs [146]. This suggests that circMyst4 may represent a novel potential therapeutic target for the treatment of PAH.

##### Others

Rutin, a plant-derived flavonoid, abundant in the human diet, has attracted significant interest owing to its potent antioxidant properties [147]. Protein kinase C alpha (PKCα), a serine/threonine kinase, plays a pivotal role in numerous cellular functions [148]. Che et al. illustrated that rutin directly bindsto PKCα via hydrogen bonding, resulting in electrostatic quenching and alterations in the protein’s secondary structure. By modulating PKCα activity, rutin effectively suppressed VSMC ferroptosis, thereby contributing to the mitigation of PAH [149]. These findings highlight the significance of small molecule–macromolecule interactions in the functional development of natural compounds and underscore the therapeutic potential of flavonoids in cardiovascular disease modulation and health maintenance.

The renin-angiotensin system (RAS) is essential for cardiovascular homeostasis and the regulation of fluid and electrolyte balance. It operates through two opposing pathways: the protective ACE2-Ang-(1-7)-Mas receptor axis and the deleterious ACE-AngII-AT1R axis [150]. Abudukeremu et al. reported that the activation of ACE2-Ang-(1-7)-Mas axis promotes PASMC ferroptosis and ROS accumulation. Moreover, the expression of proliferating cell nuclear antigen (PCNA), Vitmentin, CyclinD1 are decreased. These findings indicated that upregulation of ACE2 activity may suppress abnormal proliferation of PASMCs and help reverse hypoxia-induced pulmonary vascular remodeling [151]. These findings provide new potential therapeutic targets for the treatment of hypoxic pulmonary hypertension.

Current treatments for hypoxic pulmonary hypertension primarily aim to promote vasodilation, but they do not directly address ferroptosis in pulmonary vascular remodeling. This highlights an urgent need for strategies that can actively reverse vascular remodeling and eventually alleviate hypoxic pulmonary hypertension.

Table 6 outlines the therapeutic strategies and related mechanisms targeting VSMC ferroptosis in PAH.

**Table 6:** Ferroptosis of VSMCs in neointimal hyperplasia.

Category	Vascular disease	Cells	Treatments	Mechanism
Pulmonary vascular diseases	PAH	PASMC	hypoxia	hypoxia→STAT3→Lnc MIR210-HG→HIF-2α↑→autography-dependent ferroptosis↑→VSMC synthetic phenotype↑→HPH↑ [142]
	PAH	PASMC	hypoxia	Ca-circSCN8A↑→EP300→FUS lactylation↑→R-loop↑→SLC7A11↓→VSMC ferroptosis↑→vascular remodeling↑→PAH↑ [143]
	PAH	PASMC	hypoxia erastin	Erastin→SLC7A11↓, GPX4↓→VSMC ferroptosis↑, VSMC proliferation↓→PAH↓ [144]
	PAH	PASMC	hypoxia	hypoxia→circ-calm4↑→COMP↓→TFR1↑, NOX2↑, GPX4↓, Fe^2+^↑→VSMC ferroptosis↑→PAH↑ [145]
	PAH	PASMC	hypoxia	SE→Circ Myst4↑→DDX5→GPX4↑→Eef1a1/ACSL4↓→VSMC ferroptosis↓→PAH↓ [146]
	PAH	PASMC	hypoxia	Rutin→PKCα→VSMC ferroptosis↓→PAH↓ [149]
	PAH	PASMC	hypoxia ACE2	ACE2-Ang-(1-7)-Mas axis→ AngII/Ang1-7 imbalance→PASMC ferroptosis↑→ROS↑→PCNA,Vitmentin, CyclinD1↓→PASMC proliferation↓→PVR↓→HPH↓ [151]

↑indicates elevation or activation, ↓indicates reduction or suppression, *PAH* pulmonary arterial hypertension, *PASMC* pulmonary artery smooth muscle cells, *PKCα* protein kinase C alpha, *STAT3* signal transducer and activator of transcription 3, *HIF-2α* endothelial PAS domain protein 1, *HPH* hypoxic pulmonary hypertension, *Circ-calm4* Circ-calmodulin 4, *COMP* cartilage oligomeric matrix protein, *TFR1* transferrin receptor, *NOX2* nicotinamide adenine dinucleotide phosphate oxidase 2, *SE* superenhancer, *DDX5* DEAD-box helicase 5, *Eef1a1* eukaryotic translation elongation factor 1 alpha 1, *ACSL4* acyl-CoA synthetase long-chain family member 4, *PCNA* proliferating cell nuclear antigen, *PVR* pulmonary vascular remodeling, *ca-circSCN8A* chromatin-associated circular RNA-SCN8A, *EP300* EP300 lysine acetyltransferase, *FUS* fused in sarcoma.

## Discussion

**Fig. 3:**
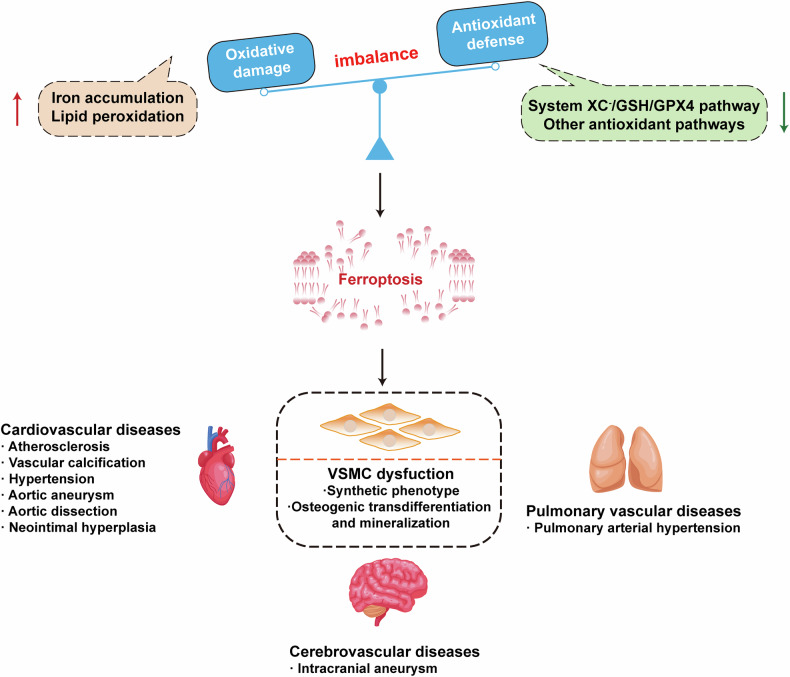
The role of ferroptosis in vascular diseases. Ferroptosis is a crucial mechanism underlying the pathogenesis of various vascular diseases, including cardiovascular diseases, cerebrovascular diseases and pulmonary vascular diseases.

Recent advances have identified ferroptosis as a crucial mechanism contributing to the pathogenesis of various vascular diseases, including cardiovascular diseases, cerebrovascular diseases and pulmonary vascular diseases (Fig. 3). Targeting ferroptosis represents a novel therapeutic approach to restore vascular homeostasis.

### Therapeutic targeting of ferroptosis in vascular diseases

A range of pharmacological agents has been developed or repurposed to inhibit ferroptosis. These include small-molecule inhibitors such as Fer-1 [35, 46, 47, 50, 51, 55, 75, 95, 97, 110, 115, 119, 134, 137, 138], liproxstatin-1 [41, 55, 98], deferoxamine (DFO) [98, 138], and N-acetylcysteine (NAC) [35, 137], as well as GPX4 activators [52] that restore cellular redox balance.

Widely used clinical drugs like metformin [107] also exhibit ferroptosis-inhibiting properties by modulating antioxidant pathways. In addition, bio-nanocomplexes containing pitavastatin can block ferroptosis-related lipid deposition and alleviate atherosclerosis in macrophages [152]. Moreover, canagliflozin inhibits endothelial cell ageing through ROS/ERK and ferroptosis pathways [153].

Additionlly, various natural compounds, such as echinatin [94], catechin [96], oleoylethanolamide [123], fisetin [115], GM3 [62], α-tocopherol [57], eugenol [49], MO [135], ginger [136], and rutin [149] have demonstrated strong potential in limiting ferroptosis by targeting key regulators like SLC7A11 and GPX4.

Recent studies have also uncovered the regulatory role of non-coding RNAs (e.g., circCDYL [60], LncMIR210HG [142], circ-calm4 [145], circMyst4 [146], chromatin-associated circ RNA SCN8A [143]) in ferroptosis, revealing opportunities for RNA-based interventions.

Furthermore, exosome-based strategies, such as GelMA-exo-MSCs [36] and MSC-derived extracellular vesicles (EVs) [46], and nanomaterial-assisted delivery systems offer promising platforms for tissue-targeted therapy.

A growing list of molecular targets, including PKD [118], PARP1 [25], Padi4 [46], HMGB2 [44], WTAP [42], METTL3 [66], SP2509 [80], BRD4770 [72], LDHA [71], KLF15 [89] and others, further expands the therapeutic landscape of ferroptosis regulation and intervention.

Several potential biomarkers for ferroptosis have promising futures for clinical application. These include lipid peroxidation products (e.g., malondialdehyde, 4-hydroxynonenal) detected in plasma [154]; EVs carrying ferroptosis-related molecules and lipid metabolites [155]; circulating iron metabolism markers, such as transferrin saturation and labile plasma iron; and molecular signatures, including GPX4 downregulation and ACSL4 upregulation in circulating cells [156].

Despite these promising developments, several critical challenges remain. First, ferroptosis displays context-dependent effects—its inhibition may protect against aneurysm formation but could aggravate neointimal hyperplasia or PAH. This duality demands disease-specific and temporally precise interventions. Second, specificity is a major concern, as ferroptosis inhibitors may affect multiple cell types, potentially interfering with physiological cell turnover or immune responses. Third, drug delivery represents difficulties, especially for targeting deep vascular tissues penetration or achieving VSMC-specific localization without affecting endothelial or immune cells. Fourth, timing is also crucial, as intervening either too early or too late in disease progression may diminish therapeutic efficacy or even cause adverse effects. Moreover, the lack of reliable biomarkers for early ferroptosis detection in clinical settings hinders personalized therapy. Finally, in terms of clinical application, ferroptosis-targeting therapies hold promise for slowing or reversing vascular degeneration, limiting aneurysm growth, and improving outcomes in post-interventional restenosis. However, limitations include the lack of human clinical trials, an incomplete understanding of long-term safety, and potential off-target effects. Furthermore, individual variability in ferroptosis sensitivity and redox metabolism poses additional barriers to standardized treatment.

### Comparative analysis of therapeutic targets

Core executors such as the GPX4–GSH/SLC7A11 and FSP1–CoQ10 pathways exert direct control over ferroptotic thresholds, enabling rapid and potent effects, which are ideal for contexts of excessive VSMC death (e.g., AA). However, their systemic modulation risks off-target redox and immune effects without targeted delivery. Conversely, tissue-specific upstream regulators (e.g., METTL3, KLF9, HMGB2, PARP1) enable context-driven modulation with higher selectivity but generally slower kinetics. In sum, executor-level targets are promising for acute or degenerative phases, while upstream regulators offer safer, precision approaches, which are especially useful in proliferative vascular diseases. A comparative evaluation of the major therapeutic modalities targeting ferroptosis is summarized in Table 7, highlighting their mechanistic precision, deliverability, and clinical translation.

**Table 7:** Comparative analysis of therapeutic modalities targeting ferroptosis in vascular diseases.

	Small-molecule modulators	RNA-based therapeutics	Natural compounds
Specificity	Target enzyme or redox system (e.g., GPX4, FSP1, iron chelators), which often have multitarget effects.	Gene-level precision through sequence complementarity (lncRNA, miRNA, circRNA). Allows pathway-specific silencing or enhancement.	Broad pleiotropic antioxidant activity; generally low target selectivity.
Deliverability	Orally or intravenously feasible; some formulations (e.g., liproxstatin-1 analogs, deferiprone) show good bioavailability.	Require carriers such as lipid nanoparticles or polymers; targeted delivery to VSMCs remains under investigation.	Orally bioavailable; stability and systemic absorption vary by compound.
Clinical translation	Deferiprone and deferoxamine are FDA-approved iron chelators under evaluation for vascular oxidative injury (Phase II). Statins and SGLT2 inhibitors exhibit anti-ferroptotic effects in cardiovascular trials.	Vascular-targeted RNA-based therapeutics for ferroptosis (circ CYDL, lncMIR210HG, and circ-calm4) are effective in animal models.	Several compounds (e.g., ginger, catechin, echinatin, and fisetin) show ferroptosis modulation in animal models.

### The dual role of ferroptosis in vascular diseases

Ferroptosis plays a context-dependent dual role in vascular pathophysiology. In certain conditions, such as AA, excessive ferroptotic death of VSMCs weakens the vascular wall, leading to structural instability and aneurysm formation. In this context, ferroptosis acts as a detrimental mechanism, contributing to tissue degeneration and vessel rupture.

Conversely, in PAH and other proliferative vascular disorders, suppression of ferroptosis allows uncontrolled VSMC proliferation, promoting neointimal hyperplasia and vascular remodeling. Here, ferroptosis serves as a protective brake against hyperproliferation.

These contrasting effects underscore the complexity of ferroptosis regulation in vascular diseases. Therapeutic modulation of ferroptosis must therefore be disease-specific and temporally precise, balancing the need to prevent pathological cell loss while restraining excessive proliferation. Understanding this duality will be crucial for developing safe and effective ferroptosis-based interventions.

### The context-dependent role of m⁶A modification in VSMC ferroptosis

Recent studies have revealed that m⁶A RNA methylation serves as a dynamic and context-dependent regulator of ferroptosis in VSMCs, orchestrated by the coordinated actions of writers, readers, and erasers.

Among the writers, METTL14 promotes ferroptosis by catalyzing m⁶A modification of ACSL4 mRNA, which is recognized by the reader IGF2BP2, enhancing transcript stability and lipid peroxidation during thoracic AA formation [41]. Similarly, WTAP acts as a cofactor to induce m⁶A methylation and stabilize BASP1 mRNA, thereby aggravating VSMC ferroptosis and promoting abdominal AA [42]. In contrast, METTL3 exerts dual and context-dependent effects: in thoracic aortic dissection, METTL3 elevation correlates with suppressed SLC7A11 and FSP1 expression, enhancing ferroptotic sensitivity [66]; however, in AA and dissection, METTL3-mediated m⁶A modification of lncRNA NORAD stabilizes the HUR/GPX4 signaling axis via YTHDF2, thereby inhibiting ferroptosis and protecting against vascular injury [68]. Furthermore, in arteriovenous fistula, METTL3/YTHDF1 enhances m⁶A modification and translation of SLC7A11, promoting VSMC proliferation and ferroptosis resistance [130].

Collectively, these findings highlight that m⁶A writers (METTL3/14, WTAP) can either promote or suppress ferroptosis depending on disease context, while readers (YTHDF1/2, IGF2BP2) determine the stability or translation of ferroptosis-related transcripts. The roles of erasers (FTO, ALKBH5) in VSMC ferroptosis remain largely unexplored, representing an important direction for future investigation.

### Cell type-specific differences in ferroptosis: the unique role of VSMCs

Ferroptosis manifests differently across cardiovascular cell types, reflecting their distinct metabolic profiles and physiological functions. VSMCs are uniquely sensitive to ferroptosis due to their high phenotypic plasticity and dynamic transitions between contractile and synthetic states. This process amplifies iron uptake and polyunsaturated lipid metabolism, rendering VSMCs highly vulnerable to oxidative stress. Ferroptotic VSMCs contribute directly to various vascular diseases, making this cell type a central driver of structural vascular pathology.

On the other hand, cardiomyocyte ferroptosis primarily arises during ischemia-reperfusion injury or doxorubicin-induced cardiomyopathy, where excessive mitochondrial ROS leads to contractile dysfunction rather than vessel wall degeneration.

Besides, endothelial cells undergo ferroptosis mainly under disturbed flow, hyperglycemia, or inflammatory stress, causing barrier disruption and endothelial-to-mesenchymal transition, which are key early events in atherogenesis.

Together, these distinctions underscore that VSMC ferroptosis represents a structurally and functionally unique form of cell death, integrating redox imbalance, phenotypic switching, and ECM remodeling. Understanding these cell-type-specific ferroptotic signatures provides a foundation for accurate therapeutic strategies in cardiovascular contexts.

## Conclusion and future perspectives

In summary, ferroptosis, a form of regulated cell death driven by iron-dependent lipid peroxidation, has emerged as a central mechanism in the pathogenesis of various vascular diseases. Recent insights highlight its pivotal role in VSMCs diseases, including cardiovascular diseases, cerebrovascular diseases, and pulmonary vascular diseases. The dysregulation of ferroptosis-related pathways, particularly those involving SLC7A11/GPX4, lipid metabolism, and oxidative stress, contributes to disease progression by promoting VSMC death, phenotypic switching, and ECM remodeling.

This review synthesizes multi-level regulatory mechanisms which govern ferroptosis in VSMCs. (1) Core executors that directly determine ferroptotic sensitivity (e.g., GPX4, FSP1); (2) Upstream regulators involving epigenetic, transcriptional, and post-translational mechanisms (e.g., METTL3, KLF9, HMGB2); (3) Cellular cross-talk and microenvironment (e.g., MSC-EV, NETs, Gelma-exo-MSC); (4) Immune and inflammatory (e.g., macrophage polarization, IL-6, Ptsg2). These layers converge on the ferroptotic machinery to govern VSMC fate, offering mechanistic insight into how ferroptosis shapes vascular remodeling and pathology.

Therapeutic modulation of ferroptosis through small molecules, RNA-based interventions, natural compounds, and nanocarrier or exosome systems holds promise for restoring vascular homeostasis and integrity.

However, despite these advances, ferroptosis remains a promising yet underexplored target in vascular biology. The complexity of its regulation, its context-dependent role in disease progression, and the challenge of achieving cell-type and disease-stage specificity necessitate a deeper mechanistic understanding. Current findings are largely limited to preclinical models, and translation into clinical applications is still in an initial phase.

Three key challenges must be addressed to enable successful translation. First, cell-type specificity: future studies should employ single-cell omics, spatial transcriptomics, and ligand-modified nanocarriers to map and manipulate ferroptotic responses in specific vascular cell types, particularly VSMCs, endothelial cells, and macrophages, to ensure selective targeting while minimizing systemic oxidative and immune side effects. Second, biomarker development: reliable biomarkers are essential for clinical translation. Integrative approaches combining plasma lipid peroxidation products, EV with ferroptosis signatures could enable dynamic monitoring of ferroptosis activity and therapeutic efficacy in vivo. Third, resolving the dual-role paradox: the context-dependent dual nature of ferroptosis, which is detrimental in degenerative conditions such as AA, but protective in proliferative diseases like PAH, necessitates stage- and tissue-specific modulation. EVs with temporally controlled drug delivery systems may help define optimal therapeutic “windows” for ferroptosis regulation.

Therefore, there is an urgent need for further mechanistic studies to elucidate the upstream regulators and downstream effectors of ferroptosis in VSMCs. Moreover, translational research is essential to evaluate safety, efficacy, and delivery strategies in humans. Such efforts will facilitate ferroptosis-targeted therapies that may ultimately transform the treatment landscape for vascular diseases.
